# High prevalence of vitamin D insufficiency and its association with obesity and metabolic syndrome among Malay adults in Kuala Lumpur, Malaysia

**DOI:** 10.1186/1471-2458-11-735

**Published:** 2011-09-27

**Authors:** Foong-Ming Moy, Awang Bulgiba

**Affiliations:** 1 Julius Centre University of Malaya, Department of Social & Preventive Medicine, Faculty of Medicine, University of Malaya, 50603 Kuala Lumpur, Malaysia

## Abstract

**Background:**

Vitamin D status, as indicated by 25-hydroxyvitamin D is inversely associated with adiposity, glucose homeostasis, lipid profiles, and blood pressure along with its classic role in calcium homeostasis and bone metabolism. It is also shown to be inversely associated with metabolic syndrome and cardiovascular diseases in western populations. However, evidence from the Asian population is limited. Therefore, we aim to study the prevalence of vitamin D insufficiency (< 50 nmol/L) and the association of 25-hydroxyvitamin D with metabolic risk factors among an existing Malay cohort in Kuala Lumpur.

**Methods:**

This is an analytical cross sectional study. A total of 380 subjects were sampled and their vitamins D status (25-hydroxyvitamin D), fasting blood glucose, full lipid profile were assessed using venous blood. Systolic and diastolic blood pressure, weight, height and waist circumference were measured following standard protocols. Socio-demographic data such as sex, age, smoking status etc were also collected. Data was analysed using t-test, chi-square test, General Linear Model and multiple logistic regression.

**Results:**

Females made up 58% of the sample. The mean age of respondents was 48.5 (SD 5.2) years. Females had significantly lower mean Vitamin D levels (36.2; 95% CI: 34.5, 38.0 nmol/L) compared to males (56.2; 95% CI: 53.2, 59.2 nmol/L). Approximately 41% and 87% of males and females respectively had insufficient (< 50 nmol/L) levels of 25-hydroxyvitamin D (p < 0.001). The prevalence of Metabolic Syndrome for the whole sample was 38.4 (95% CI: 33.5, 43.3)%. In the multivariate model (adjusted for age, sex, abdominal obesity, HDL-cholesterol, diastolic blood pressure), insufficient Vitamin D status was significantly associated with 1-year age increments (OR: 0.93; 95% CI: 0.88, 0.98), being female (OR: 8.68; 95% CI: 5.08, 14.83) and abdominal obesity (OR: 2.57; 95% CI: 1.51, 4.39). Respondents with insufficient vitamin D were found to have higher odds of having Metabolic Syndrome (OR: 1.73; 95% CI: 1.02, 2.92) after adjusting for age and sex.

**Conclusions:**

Our results highlight the high prevalence of vitamin D insufficiency among Malay adults in Kuala Lumpur. Vitamin D insufficiency is independently associated with younger age, female sex and greater abdominal obesity. Vitamin D insufficiency is also associated with Metabolic Syndrome.

## Background

Clinically, vitamin D has an established role in calcium and bone metabolism. Recently, vitamin D insufficiency has been shown to be associated with increased risk of developing type 2 diabetes mellitus and cardiovascular disease (CVD), as well as with cardiovascular risk factors such as hypertension and obesity [1]. Systematic reviews [2-4] suggest a possible inverse association between Vitamin D and cardiovascular risks. In a meta-analysis by Parkera et al [5], individuals with the highest levels of serum vitamin D were associated with a 43% reduction in cardio-metabolic disorders (OR 0.57, 95% CI: 0.48-0.68).

Vitamin D is synthesized in the skin after sunlight exposure or can be obtained through a balanced dietary intake. However, it is well known that natural sources of vitamin D in foods are not sufficient to supply the normal body requirements. Therefore, skin synthesis of vitamin D through exposure to sunlight is thought to constitute the major source of vitamin D [6]. This significant role of sunlight in vitamin D synthesis suggests a low prevalence of vitamin D deficiency in tropical countries. However, studies carried out in Hawaii, Turkey, India, Iran and Saudi Arabia had shown a high prevalence of vitamin D deficiency [7-11] with the respondents' behavior of avoiding sunlight or clothing which prevented sunlight exposure being the reasons for this phenomenon.

Malaysia is a tropical country located at the Equator and is sunny all year round. However, the information on vitamin D status of the adult population is scarce. To date, the only available Malaysian studies were focused on the association of vitamin D status and bone health among child-bearing [12] and post-menopausal women [13]. These studies did not particularly focus on vitamin D and cardiometabolic risks. Recent evidence show possible associations of vitamin D status with metabolic syndrome, diabetes, cardiovascular diseases and cancers in the long term [1-3,14,15]. This may increase the incidence of metabolic syndrome and these chronic diseases in the future. In this study, we set out to look at the vitamin D status of a Malay cohort in Kuala Lumpur; and to demonstrate the association of metabolic risk factors with vitamin D status.

## Methods

### Study design & sampling method

This was an analytical cross-sectional study which was part of a voluntary health screening program in a public university in Kuala Lumpur. Universal sampling was conducted where all employees aged 35 years and above were invited to participate. From this population, a convenient sample of 380 Malay employees agreed to participate in our study to assess their vitamin D status. The sample size of 380 was constrained by our limited research budget.

### Study population

The majority (70%) of employees in this public university are of Malay ethnicity. They are Muslims by religion; tend to have darker skin and most of the females adopt concealing dressing style such as veils (head scarves, face not covered), long sleeves, long skirts etc. Most of the employees in this university work indoors.

### Ethics consideration

Approval was obtained (Reference Number: MEC 782.18) from the Medical Ethics Committee of the Medical Faculty which is responsible for ethical issues in all research projects involving humans conducted by Medical Faculty staff of the university. Approval was also obtained from the management of the university. Written informed consent was given by all participants.

### Data Collection

Data was collected during the health screening from May to July 2010 in the university campus. Vitamin D status of the participants was assessed using venous blood. Vitamin D adequacy was evaluated by measuring serum 25-hydroxyvitamin D (25 (OH) D) concentration, as this was the primary circulating form of vitamin D. This serum concentration of 25(OH)D is a good reflection of cumulative exposure to sunlight and dietary intake of vitamin D, and is widely regarded as a robust ''gold standard" indicator of vitamin D status [16]. The biochemical test used was the LAISON(R) 25 OH Vitamin D TOTAL Assay. This assay used chemiluminescent immunoassay (CLIA) technology for the quantitative determination of 25(OH)D. These biochemical analyses were outsourced to a private laboratory with international quality control Certificate of Accreditation (MS ISO 15189).

There are a few categories of Vitamin D status according to circulating 25 OH Vitamin D [17]. The categories are (i) deficiency (< 25 nmol/l) where clinical alterations such as rickets, osteomalacia, myopathy, calcium malabsorption, severe hyperparathyroidism, impaired immune and cardiac function happen; (ii) insufficiency (25 - 49.9 nmol/l) where reduced bone mineral density, impaired muscle function, low intestinal calcium absorption rates, elevated PTH levels occur; (iii) hypovitaminosis (50 to 99.9 nmol/l) where slightly elevated PTH levels occur; (iv) adequacy (100 nmol/l) with no disturbances of vitamin D dependent functions and (v) intoxication when the level of 25 OH Vitamin D exceeds 250 nmol/l. In our study, subjects with 25 OH Vitamin D levels less than 50 nmol/L or 20 ng/ml were classified as having insufficient Vitamin D [17]. (To convert 1 nmol/L to 1 ng/ml is to divide by 2.5.)

Resting blood pressure was measured using a clinically validated digital automatic blood pressure monitor (Omron HEM - 907 model) while fasting lipid profile was analyzed using the Dimension^® ^clinical chemistry system which was an in-vitro diagnostic test. All biochemical analysis was conducted by the Clinical Diagnostic Laboratory of the medical centre from the same university.

Anthropometric measurements such as weight, height and waist circumference were also taken. Weight and height were measured using calibrated digital weighing scales and stadiometers respectively. The waist and hip circumferences were measured with a circumference measurement tape. The waist was defined as the point midway between the iliac crest and the costal margin (lower rib); while the hip circumference was defined as being the widest circumference over the buttocks and below the iliac crest [18,19]. All measurements were conducted by trained staff and quality checks were conducted regularly.

Body Mass Index (BMI) was derived following the formula of weight in kg/height^2 ^in meters. Overweight was defined using the Asian BMI range of 23.0 to 26.9 kgm^2 ^while obesity was defined as having a BMI ≥ 27 kgm^2 ^[20]. Metabolic Syndrome was defined according to the modified NCEP criteria [21], where the presence of any three of the following five factors is required for a diagnosis of Metabolic Syndrome: abdominal obesity (waist circumference > 90 cm in men and > 80 cm in women), hypertriglyceridaemia (triglycerides ≥ 1.7 mmol/L); low HDL cholesterol (HDL cholesterol ≤ 1.03 mmol/L for men and ≤ 1.29 mmol/L for women); elevated blood pressure (systolic blood pressure ≥ 130 mmHg and/or diastolic blood pressure ≥ 85 mmHg or current use of antihypertensive drugs); impaired fasting glucose (fasting plasma glucose ≥ 5.6 mmol/L). A short questionnaire enquiring socio-demographic characteristics, medical history of diabetes mellitus and hypertension (self-reported as diagnosed by medical doctors) was self-administered.

### Data Analysis

Data was entered and analysed using SPSS for Windows version 16.0. Categorical variables were presented as frequencies and percentages while quantitative variables were presented as mean ± standard deviation (sd) or 95% confidence interval (CI) where appropriate. Independent t test and Chi square test were used in univariate analysis. The General Linear Model (GLM) was used to adjust for gender differences on quantitative variables. Multiple logistic regression was conducted by including risk factors with p < 0.2 into the model to predict the risk factors significantly associated with vitamin D insufficiency. If both BMI and waist circumference were found to be significant, the one with the smaller p value will be selected as both are indicators of obesity and will produce collinearity if both are included in the model.

## Results

A total of 380 Malay employees participated in this study with 58.4% being females. Their mean age was 48.5 ± 5.2 years with males being significantly older (Table 1). Ninety five participants were hypertensive. Of these, 90.5% of them were currently on treatment, while forty one of them were diabetics with 82.9% on treatment. There was no significant difference (p > 0.05) in the systolic (147.4 ± 20.5 mm Hg vs 147.7 ± 13.3 mm Hg respectively) and diastolic blood pressures (89.6 ± 11.8 mm Hg vs 88.4 ± 5.5 mm Hg respectively) among the treated and untreated hypertensive participants. Similarly, no significant difference was observed in the fasting blood glucose of treated (10.2 ± 3.9 mmol/l) and untreated (9.9 ± 4.0 mmol/l) diabetic participants. About 10% of them were current smokers who were all males. Only one third of the participants had sufficient levels of vitamin D (≥ 50 nmol/L). More than 80% of participants were overweight or obese. Approximately 40% of the participants were diagnosed with Metabolic Syndrome. There were no gender difference observed in hypertension, diabetes mellitus, BMI status and metabolic syndrome except for current smokers (100% males) and insufficiency of vitamin D where there were more females (87%) had insufficient levels of 25 OH Vitamin D compared to males (41%)(p < 0.001).

**Table 1:** Baseline characteristics of participants by gender

Characteristics		Total (n = 380) n (%)	Male (n = 158) n (%)	Female (n = 222) n (%)	p value
Diabetes mellitus		41 (10.8)	19 (12.0)	22 (9.9)	0.51§
Hypertension		95 (25.0)	45 (28.5)	50 (22.5)	0.19§
					
Current smoker		37 (9.7)	37 (100)	0 (0)	**< 0.001**§
					
BMI status:	Normal weight	60 (15.8)	29 (18.4)	31 (14.0)	0.12§
	Overweight	127 (33.4)	59 (37.3)	68 (30.6)	
	Obese	193 (50.8)	70 (44.3)	123 (55.4)	
25 OH Vit D Insufficiency (< 50 nmol/l)		258 (67.9)	65 (41.1)	193 (86.9)	**< 0.001**§
Metabolic syndrome		146 (38.4)	64 (40.5)	76 (34.2)	0.21§
		(mean ± s.d.)	(mean ± s.d.)	(mean ± s.d.)	
		
Age (years)		48.5 ± 5.2	49.6 ± 5.7	47.7 ± 4.6	**< 0.001**‡
BMI (kg/m^2^)		27.5 ± 4.6	26.7 ± 4.3	28.1 ± 4.8	**0.04**‡
Waist circumference (cm)		87.5 ± 10.4	90.9 ± 9.9	85.0 ± 9.9	**< 0.001**‡
Systolic blood pressure (mm Hg)		133.7 ± 18.7	136.2 ± 15.8	132.0 ± 20.4	**0.03**‡
Diastolic blood pressure (mm Hg)		82.1 ± 11.5	83.4 ± 10.5	81.1 ± 12.1	0.06‡
Fasting blood glucose (mmol/l)		5.9 ± 2.4	5.9 ± 2.1	5.9 ± 2.6	0.95‡
Triglycerides (mmol/l)		1.6 ± 0.9	1.9 ± 0.9	1.4 ± 0.7	**< 0.001**‡
Total cholesterol (mmol/l)		5.4 ± 0.9	5.5 ± 0.9	5.4 ± 0.9	0.44‡
HDL-cholesterol (mmol/l)		1.3 ± 0.3	1.2 ± 0.2	1.4 ± 0.3	**< 0.001**‡
LDL-cholesterol (mmol/l)		3.3 ± 0.9	3.4 ± 0.8	3.3 ± 0.8	0.24‡
25 OH Vitamin D (nmol/l)		44.5 ± 18.7	56.2 ± 19.0	36.2 ± 13.4	**< 0.001**‡

‡t test, §chi-square test

The metabolic risk factors are also presented in Table 1. Males were found to have significantly higher readings in waist circumference, systolic and diastolic blood pressure, triglycerides and 25 OH Vitamin D levels compared to their counterpart (p < 0.05). On the other hand, females had significantly higher mean BMI and HDL-cholesterol levels than males (p < 0.05). There was no difference in fasting blood glucose, total cholesterol and LDL-cholesterol levels between the sexes (p > 0.05).

Table 2 displays the comparison between the sufficient and insufficient vitamin D groups adjusted by gender. The vitamin D insufficient group was significantly younger (p < 0.001). Obesity as reflected by BMI and waist circumference was strongly associated with Vitamin D status (p < 0.001). The mean BMI and waist circumference was significantly (p < 0.001) higher in the vitamin D insufficient group. There was no significant association between vitamin D insufficiency with systolic and diastolic blood pressure, HDL-cholesterol and glucose except for triglycerides, which was significantly higher in the vitamin D insufficient group (p = 0.018). However, significant association was found between vitamin D levels and total metabolic risk scores. Those with vitamin D insufficiency had higher metabolic risk scores (p = 0.009).

**Table 2:** Comparison of metabolic risk factors according to Vitamin D status

	Vitamin D Insufficiency		
	
Metabolic risk factors	Yes (< 50 nmol/L) n = 258 **mean ± s.d**.	No (≥ 50 nmol/L) n = 122 **mean ± s.d**.	GLM p-value
Age (years)	47.77 ± 4.68	49.95 ± 5.82	**0.007**
BMI (kg/m^2^)	28.25 ± 4.64	25.96 ± 4.27	**< 0.001**
Waist circumference (cm)	87.78 ± 10.34	86.80 ± 10.44	**< 0.001**
Systolic blood pressure (mm Hg)	133.16 ± 19.04	134.98 ± 18.12	0.915
Diastolic blood pressure (mm Hg)	81.98 ± 11.74	82.24 ± 11.08	0.518
Glucose (mmol/l)	5.99 ± 2.70	5.69 ± 1.67	0.303
Triglyceride (mmol/l)	1.59 ± 0.86	1.64 ± 0.87	**0.018**
HDL-cholesterol (mmol/l)	1.38 ± 0.31	1.27 ± 0.26	0.714
Total metabolic risk score	2.2 ± 1.4	1.9 ± 1.3	**0.009**

s.d. - standard deviation

Table 3 shows the crude and adjusted Odds Ratio (OR) of metabolic risk factors with vitamin D status. In the crude analysis; age, female, BMI, waist circumference and HDL-cholesterol were found to be significantly associated with vitamin D insufficiency (< 50 nmol/l). In the multivariate model (adjusted by age, sex, waist circumference, diastolic blood pressure and low HDL-cholesterol); only age, sex and waist circumference remained significant (p < 0.05). Older age participants were found to have lower odds of having vitamin D insufficiency, while females had 8.68 (95% CI: 5.08, 14.83) times odds of having vitamin D insufficiency. Participants who were obese (indicated by central obesity) too had significantly higher odds (2.57; 95% CI: 1.51, 4.39) of having vitamin D insufficiency. Other metabolic risk factors were not statistically associated with vitamin D status. In a separate model adjusted by age and sex, participants with vitamin D insufficiency had significantly higher odds of having metabolic syndrome (1.73; 95% CI: 1.02, 2.92).

**Table 3:** Comparison of crude and adjusted risks of age, gender and metabolic factors with Vitamin D Insufficiency

	Vitamin D Insufficiency			
	
	Crude OR (95% CI)	P	Adjusted OR (95% CI)	P
Age	0.92 (0.88, 0.96)	**< 0.001**	0.93 (0.88, 0.98)*	**0.007**
Female	9.52 (5.76, 15.74)	**< 0.001**	8.68 (5.08, 14.83)*	**< 0.001**
Overall obesity (BMI)	1.94 (1.10, 3.41)	**0.021**	-	
Abdominal obesity	2.81 (1.80, 4.39)	**< 0.001**	2.57 (1.51, 4.39)*	**0.001**
High systolic blood pressure	0.86 (1.56, 1.32)	0.494	-	
High diastolic blood pressure	1.35 (0.86, 2.14)	0.195	1.53 (0.87, 2.69)*	**0.137**
Hyperglycaemia	1.05 (0.64, 1.71)	0.853	-	
Hypertriglyceridemia	0.88 (0.57, 1.38)	0.588	-	
Low HDL-cholesterol	1.97 (1.18, 3.31)	0.010	1.03 (0.58, 1.91)*	0.923
Metabolic syndrome	1.29 (0.82,2.02)	0.271	1.73 (1.02, 2.92)**	**0.044**

*Adjusted for age, sex, abdominal obesity, diastolic blood pressure, Low HDL-cholesterol

** Adjusted for age and sex

## Discussion

The serum 25-hydroxyvitamin D, 25(OH)D level for optimal health appears to be in the range of 100-150 nmol/L [5,22]. Our findings demonstrated a high proportion (approximately 70%) of participants with insufficient vitamin D level (< 50 nmol/L or 20 ng/ml). In many countries throughout all continents, vitamin D insufficiency (< 50 nmol/L) exists in around 50% of the populations [23]. Within the Asian region, there are not many reports on vitamin D status of the population except pockets of studies among children, pregnant women or post-menopausal women. In India, generally low serum 25(OH)D concentrations have been reported. The mean serum 25(OH)D was 30 nmol/L in a survey among hospital staff from India[24], while the mean serum 25(OH)D was 35 nmol/L among pregnant women [25] and 36 nmol/L in postmenopausal women [26]. In Beijing, China, mean serum 25(OH)D in adolescent girls was 30 - 36 nmol/L [27]. In Japan, a low mean serum 25(OH)D of 34 nmol/L was observed in women younger than 30 years and 30 nmol/L in immobile older persons [28]. In the Korea National Health and Nutrition Examination Survey (KNHANES), Vitamin D insufficiency (< 50 nmol/l) was found in 47.3% males and 64.5% females [29].

In Malaysia, there are few published reports on vitamin D status among the adult population. One published study reported the vitamin D levels of post-menopausal women [13] and another studied on women of child bearing age [12]. The vitamin D status of women from both these studies was better than our female participants. The mean vitamin D level of postmenopausal Malay women in the study by Rahman et al [13] was 44.4 ± 10.6 nmol/L, which was higher than our female participants. Green et al [12] reported over 60% of Malaysian women had vitamin D levels below 50 nmol/L compared to ours of approximately 87%.

Our female participants had significantly lower mean vitamin D levels than males after adjustment for age, central obesity, HDL-cholesterol and diastolic blood pressure. Similar results have been reported elsewhere [7,8,11,23]. Other possible reasons contributing to this difference could also be explained by their clothing style (wearing long sleeves, long skirts and veil) which is culturally or religiously related (results not shown). There have been no published findings on vitamin D levels among Malaysian men and not many such studies in other countries. Our male participants' vitamin D status are comparable with the Korean males (43% with Vitamin D insufficiency) [29], poorer than the Vietnam males (20% with Vitamin D insufficiency) [30] but much better than males from Middle Eastern countries like Iran (69% with vitamin D deficiency) [8].

Existing evidence show that elderly individuals are more likely to have low vitamin D levels [11,23]. However, we found age to be negatively associated with vitamin D status, where older participants had higher vitamin D levels. The reason for this result remains unclear. Possibly, the age of our working participants was not representative of the general population where there were no elderly participants. However, this finding concurs with that from the national study from Korea [29] which found Vitamin D insufficiency most prevalent in the younger age groups and least prevalent in the age group of 50 s and above. Further studies are needed to investigate this paradoxical finding as it would appear more logical to have it the other way around.

Abdominal obesity was significantly associated with vitamin D status; similar to studies reported elsewhere [31-34]. This may be due to vitamin D being soluble in fat which is largely sequestered in adipose tissue and is therefore low in serum among obese individuals [33,35]. This will give rise to reduced bioavailability of vitamin D metabolite which regulates transcription of multiple gene products with antiproliferative, prodifferentiative, and immunomodulatory effects [1]. Consequently, these obese individuals will be more susceptible to metabolic syndrome [36-38] as well as cardiovascular diseases [1-3,17].

Contrary to existing evidence, our findings did not show significant associations among individual metabolic risk factors such as fasting blood glucose, triglycerides, HDL cholesterol and blood pressure with Vitamin D status. Our small sample size may not have adequate power to detect significant association within individual risk factors. On the other hand, participants with insufficient vitamin D status had higher odds for metabolic syndrome after adjustment for age and sex. As reported elsewhere [5,36,39,40], having vitamin D insufficiency was independently associated with an increased risk of having metabolic syndrome. Our findings concur with those in Western countries.

These findings have given rise to concerns about the vitamin D status of the population in general and its adverse effects on health. Urgent measures needed should include the assessment of vitamin D status among the population, more particularly in females. Public health messages on the importance of vitamin D, ways of achieving optimal vitamin D status and associated cardio-metabolic risks should also be disseminated to the public.

However, these results may not be generalizable to the entire Malaysian population as our participants were urban Malays engaged in essentially indoor-based occupations. We were also not able to measure the parathyroid hormone (PTH) of these participants. PTH should be included as a functional index of vitamin D status in future studies. Other factors associated with low vitamin D status such as dietary survey or physical activity pattern should also be included in future studies. Comparison of vitamin D status between studies is difficult as the definition of vitamin D insufficiency and deficiency, as well as assay methodology of 25(OH)D vary between studies. It is also noted that the sample size of this study may have inadequate power to detect association of vitamin D status with some metabolic risk factors.

On the other hand, this study remains as one of a few studies on the vitamin D status and its association with obesity and metabolic syndrome for both males and females in Malaysia. This study is also timely as the population has high prevalence of obesity [41]. Although this study was confined to only Malay participants, this could serve as a pilot study for future studies that involve all races of the country.

## Conclusions

This study highlights the high prevalence of vitamin D insufficiency among Malay adults especially females in Kuala Lumpur. Vitamin D insufficiency is associated with younger age, gender (female) and greater abdominal obesity. Vitamin D insufficiency is also associated with Metabolic Syndrome. Our findings concur with those from the West.

## Competing interests

The authors declare that they have no competing interests.

## Authors' contributions

FMM contributed to conceptualizing the paper, data entry, data analysis and writing of the manuscript while AB contributed in data analysis and writing of the manuscript. Both authors approved the final draft.

## Pre-publication history

The pre-publication history for this paper can be accessed here:

http://www.biomedcentral.com/1471-2458/11/735/prepub
